# Binding Mode Prediction of Evodiamine within Vanilloid Receptor TRPV1

**DOI:** 10.3390/ijms13078958

**Published:** 2012-07-18

**Authors:** Zhanli Wang, Lidan Sun, Hui Yu, Yanhui Zhang, Wuzhuang Gong, Hongwei Jin, Liangren Zhang, Huaping Liang

**Affiliations:** 1 State Key Laboratory of Trauma, Burns, and Combined Injury, Department 1, Research Institute of Surgery, Daping Hospital, The Third Military Medical University, Chongqing 400042, China; E-Mail: wang.zhanli@hotmail.com; 2 The First Affiliated Hospital, Baotou Medical College, Baotou 014010, China; E-Mails: yanhui_zhang1200@sina.com (Y.Z.); gongwz73@sina.com (W.G.); 3 State Key Laboratory of Natural and Biomimetic Drugs, Peking University, Beijing 100191, China; E-Mails: sld85@126.com (L.S.); jinhw@bjmu.edu.cn (H.J.); 4 The Second Affiliated Hospital, Baotou Medical College, Baotou 014010, China; E-Mail: huiyu2008@hotmail.com

**Keywords:** transient receptor potential vanilloid type 1, homology modeling, molecular docking, molecular dynamics, capsaicin, evodiamine

## Abstract

Accurate assessment of the potential binding mode of drugs is crucial to computer-aided drug design paradigms. It has been reported that evodiamine acts as an agonist of the vanilloid receptor Transient receptor potential vanilloid-1 (TRPV1). However, the precise interaction between evodiamine and TRPV1 was still not fully understood. In this perspective, the homology models of TRPV1 were generated using the crystal structure of the voltage-dependent shaker family K^+^ channel as a template. We then performed docking and molecular dynamics simulation to gain a better understanding of the probable binding modes of evodiamine within the TRPV1 binding pocket. There are no significant interspecies differences in evodiamine binding in rat, human and rabbit TRPV1 models. Pharmacophore modeling further provided confidence for the validity of the docking studies. This study is the first to shed light on the structural determinants required for the interaction between TRPV1 and evodiamine, and gives new suggestions for the rational design of novel TRPV1 ligands.

## 1. Introduction

Transient receptor potential vanilloid-1 (TRPV1), a calcium ion channel activated by vanilloids, protons, heat and several inflammatory mediators, has been implicated as a pain-sensing transducer and plays a key role in the maintenance of inflammatory conditions in a variety of diseases and injury states [1]. TRPV1 is a 95 kDa protein consisting of 838 amino acids. This receptor possesses 6 transmembrane domains and intracellular *N*-and *C*-terminals [2]. Most probably, TRPV1 forms tetramers in the membrane and is sensitive to a variety of chemicals, such as capsaicin, resiniferatoxin, anandamide, olvanil, and evodiamine [3–7]. Recent investigations aim to identify TRPV1 regions involved in capsaicin recognition and several important amino acid residues of TRPV1 have been identified. For example, Tyr511 and Ser512 located between the second and third transmembrane segments are critical for capsaicin binding [8]. Additionally, studies have shown that TRPV1 presents a species-specific feature since rat and human TRPV1 is sensitive to capsaicin whereas rabbit TRPV1 is not sensitive [9]. Although an X-ray crystal structure has not been reported as yet, we should note that several research groups have attempted modeling TRPV1 [9–11]. These models can be used to define the structural basis for TRPV1 recognition by capsaicin or other ligands. The precise molecular basis for species-specificity of capsaicin toward the different TRPV1 models was also proposed.

**Figure 1:**
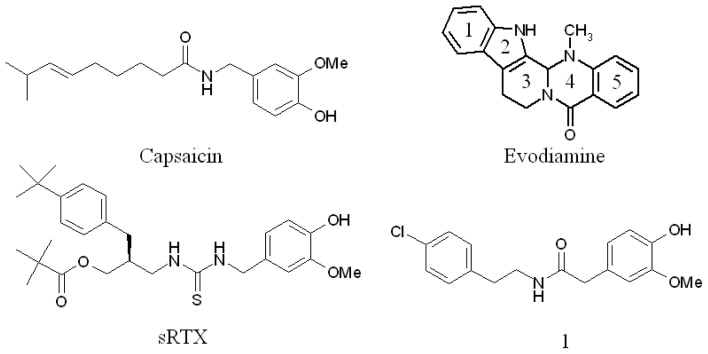
Structures of capsaicin, evodiamine, sRTX and compound 1.

Evodiamine (Figure 1), the major bioactive alkaloid isolated and purified from the Chinese herbal drug named *Evodia rutaecarpa* (Wu-Chu-Yu) [12], acts as an agonist for TRPV1 [4]. A study found that evodiamine could activate human TRPV1, which plays a fundamental role in pain and involves in the protective effects on cardiovascular and gastrointestinal systems [13]. Additionally, researchers found that evodiamine was an agonist for TRPV1 in rat and guinea-pig [4,14–16]. Moreover, Chiou *et al*. reported that evodiamine possessed a potent corporal relaxing effect in rabbit [17]. In fact, corporal relaxing effect was probably partly due to TRPV1 activation [18]. Though effects of evodiamine on TRPV1 activation in the different species had been experimentally assessed, to the best of our knowledge, there is no detailed knowledge of the binding features of evodiamine to TRPV1 up to date.

In this context, we constructed homology models of rat, human and rabbit TRPV1. Then, the specific interaction of evodiamine with TRPV1 was investigated using computational approaches. Furthermore, the interspecies differences in ligand binding were characterized. This knowledge is important to understand the molecular basis for the recognition of evodiamine by TRPV1. Moreover, the results obtained from this study will be useful in the design of novel potent TRPV1 ligands.

## 2. Results and Discussion

### 2.1. Building Rat, Human and Rabbit TRPV1 Homology Models

**Figure 2:**
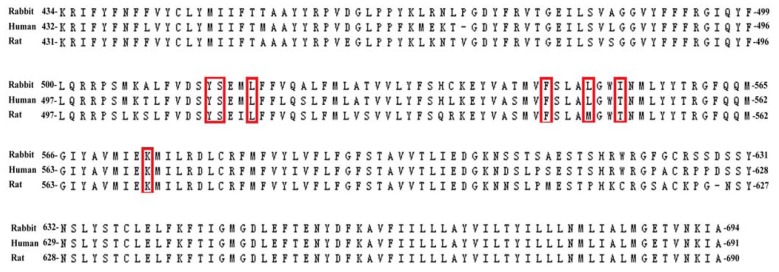
Sequence alignment of rabbit, human and rat Transient receptor potential vanilloid-1 (TRPV1).

To construct the homology models, multiple sequence alignment of TRPV1 transmembrane region for rat, human and rabbit was carried out (Figure 2). The sequence identities between the different species were greater than 88%. Moreover, the sequence alignment produced sequence similarities of greater than 93%. The rat TRPV1 residues Tyr511, Ser512, Leu515, Phe543, Met547, Thr550 and Lys571, which have been characterized by mutagenesis and docking studies and are known to influence capsaicin binding [11], are highlighted in red in Figure 2. These residues are well conserved except that Met547 is replaced with Leu in human and rabbit and Thr550 is replaced with Ile in rabbit.

**Figure 3:**
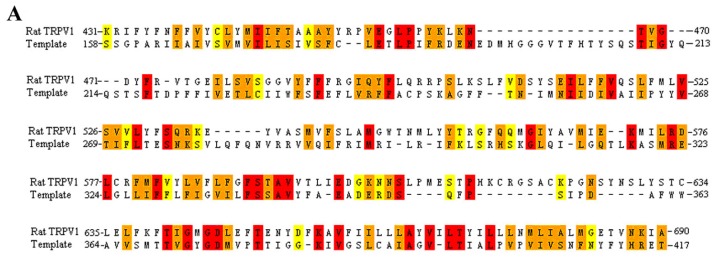

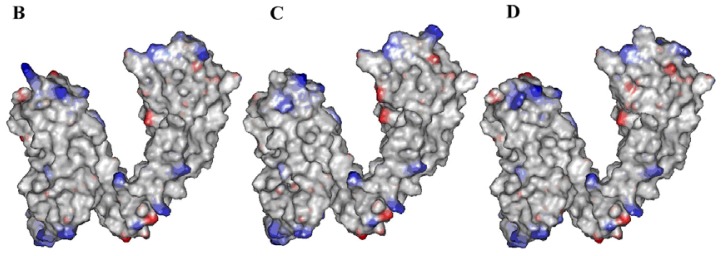
(**A**) Sequence alignment of rat TRPV1 and the voltage-dependent shaker family K^+^ channel (PDB code: 2R9R). The identical, strongly conserved, and weakly conserved residues are denoted, respectively, with red, orange, and yellow boxes. (**B**–**D**) Homology models for rat TRPV1 (**B**), human TRPV1 (**C**) and rabbit TRPV1 (**D**). Models are represented as a molecular surface colored by electrostatic potential. The color ramp for electrostatic potential ranges from red (most negative) to blue (most positive).

It is well known that the structural organization of TRPV1 is similar to that of voltage-dependent channels [19]. To date, several structures of voltage-dependent channels are available, including the X-ray crystal structure of the voltage-dependent shaker family K^+^ channel (PDB: 2R9R). The sequence similarity between rat TRPV1 and the voltage-dependent shaker family K^+^ channel is low. However, they all contain the full six transmembrane helices. Therefore, the voltage-dependent shaker family K^+^ channel was selected for generating a homology model of rat TRPV1. ClustalW program was used for identifying regions of similarity between two sequences. The automatic alignment was then manually refined to ensure the correct alignment of important functional residues (Figure 3a). The initial rat TRPV1 model was built. Then, the model was embedded into an explicit phosphatidyl oleoyl phsophatidylcholine (POPC) membrane and further refined using a 5 ns molecular dynamics (MD) simulation. The human and rabbit TRPV1 homology models were built starting from the rat model, and the initial models were then energetically refined using the same method as for rat TRPV1 model. Figure 3b–d showed the refined TRPV1 monomer models that are represented as a molecular surface colored by electrostatic potential. The quality of the refined homology models were assessed using the PROCHECK and ERRAT programs. The Ramachandran plots of the template X-ray crystal structure and refined TRPV1 monomer models were showed in Supplementary information (Figure S1). The template and our refined models for rat, human and rabbit TRPV1 yielded ERRAT scores of 94.80, 90.01, 88.32 and 88.23, respectively. These results indicated that our refined homology monomer models for rat, human and rabbit TRPV1 reached an acceptable level of accuracy. Following the development and refinement of TRPV1 monomer models, we constructed rat, human and rabbit TRPV1 tetramer models using the previously described modeling protocol [20]. Since the functional TRPV1 is a homotetramer, the constructed tetramer model might be helpful for the deep understanding of the interaction between TRPV1 and ligands.

### 2.2. Identification of Ligand-Binding Region in TRPV1

Following development and refinement of the model, we first analyzed rat TRPV1 homology model to characterize the ligand binding pocket. Three active sites were obtained using the Binding Site tools in Discovery studio software, and the locations of the three sites in the 3D structure of rat TRPV1 homology model are shown in Supplementary Information (Figure S2). The previous mutation studies suggested that the affinity of TRPV1 agonists seemed to be mainly influenced by the presence of Tyr511, Ser512, Leu515, Phe543, Met547, Thr550, and Lys571 [11]. Additionally, Blumberg *et al*. found a consistent pattern of overlap between evodiamine and TRPV1 agonists. They also found that capsazepine, a competitive antagonist of capsaicin, could inhibit the action of evodiamine in a competitive manner [4]. A rationale for this competitive mechanism is that agonists and antagonists compete for the same binding site on the receptor. It is obvious that the site 3 (red region) in this study is in agreement with the conclusion drawn by the experimental results described above. This site in our model is more compact, surrounded mainly by the residues Tyr511, Leu515, Thr550, Tyr554, Tyr555, Ile564, Tyr565, and Val567-Lys571. Leu515 lies roughly at the center of the pocket. The side chains of these amino acids are all pointing toward the binding pocket, implying structurally their direct involvement in ligand binding. Based on the experiment and our theoretical predicted results, this site is therefore chosen as the more favorable binding site to dock the ligand. However, it is noteworthy to mention that some of the residues (Ser512, Phe543 and Met547) do not seem to be part of the site 3. In order to determine whether these residues are necessary for evodiamine binding within TRPV1 models, the binding site was defined as all residues of the target within 10 Å from Leu515 for expanding the site 3, so that residues Ser512, Phe543 and Met547 were also involved in the binding site.

### 2.3. Detection of Binding between TRPV1 Homology Models and Evodiamine

In order to attempt to set up a reliable theoretical method to fully understand the interaction mode between TRPV1 and evodiamine, capsaicin was firstly docked into rat, human and rabbit TRPV1 models to validate the docking method. The models of TRPV1-capsaicin complex were then embedded into a POPC membrane, and a 5 ns MD simulation was performed to investigate the binding modes between TRPV1 models and capsaicin (Figure S3). The predicted docking orientation of the capsaicin was in excellent agreement with other studies [11]. It is worth note that the residue at position 550 (rat and human) and 553 (rabbit) located in the upper of the ligand binding area appears to directly impact capsaicin binding. In rabbit TRPV1, this amino acid is substituted with a bulkier residue Ile553, which could decrease the volume of the binding pocket and disturb the binding of the nonenyl tail of capsaicin. Moreover, the binding energy values obtained for capsaicin binding in rat and human models were −4.07 and −4.09 kcal/mol, respectively. In contrast, the value obtained with the rabbit model was −2.33 kcal/mol, indicating a decreased binding affinity compared with the other two species investigated. The binding energy values are in agreement with the biological activity data reported [9]. Thus, our results confirmed that TRPV1 homology models were reasonable, and the docking method was reliable for the determination of the interactions between TRPV1 and ligands.

**Figure 4:**
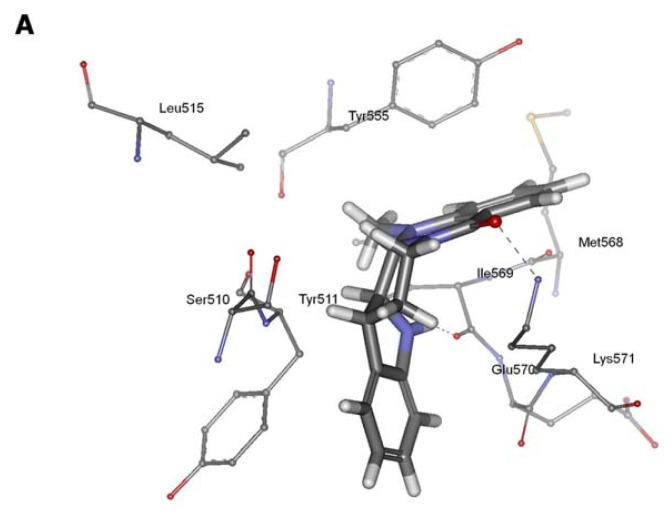

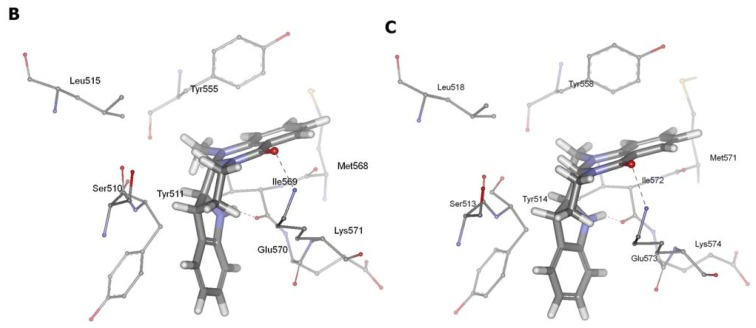
Binding of evodiamine to the active sites of rat (**A**), human (**B**) and rabbit (**C**) TRPV1 models.

Based on this model, molecular docking was used to investigate the binding modes of evodiamine with rat TRPV1. The result showed that evodiamine occupied binding pocket formed by Ser510, Tyr511, Leu515, Tyr555, Met568, Ile569, Glu570 and Lys571 (Figure 4a). The ring 1 of evodiamine pointed toward Tyr511, establishing a hydrophobic interaction. The ring 5 of evodiamine occupied a small cavity in which it formed π–π stacking and hydrophobic interactions with Tyr555 of rat TRPV1. In addition, the indole nitrogen and formyl carbonyl oxygen atoms of evodiamine made H-bonding interactions with Ile569 and Lys571, respectively (Figure 4). The binding energy calculations generated values of −3.87 kcal/mol, which was slightly higher than this for capsaicin, indicating a little decrease binding affinity compared with capsaicin. This docking result was in accordance with the experimental biological data for rat TRPV1 [4].

Furthermore, evodiamine was docked into human and rabbit TRPV1 models to investigate any interspecies differences in their respective binding modes. Our results showed that evodiamine docked into both species binding pockets in a similar orientation. The ring 1 of evodiamine pointed toward Tyr511 (human) and Tyr514 (rabbit), establishing a hydrophobic interaction. The ring 5 of evodiamine pointed toward Tyr555 (human) and Tyr558 (rabbit), forming aromatic π–π interactions. In addition, evodiamine made two H-bonds between the formyl carbonyl oxygen and the side chains of Lys571 (human) and Lys 574 (rabbit) and between the indole nitrogen and the side chains of Ile569 (human) and Ile572 (rabbit) (Figure 4). The values obtained for evodiamine binding in human and rabbit TRPV1 models were −3.76 and −3.70 kcal/mol, respectively, confirming that evodiamine had similarly binding affinity in different species investigated. It is well known that TRPV1 showed differences in capsaicin sensitivity in a rat model as compared with a rabbit model. However, our results suggested that TRPV1 did not demonstrate any species-specific sensitivity to evodiamine.

### 2.4. Pharmacophore Studies

**Figure 5:**
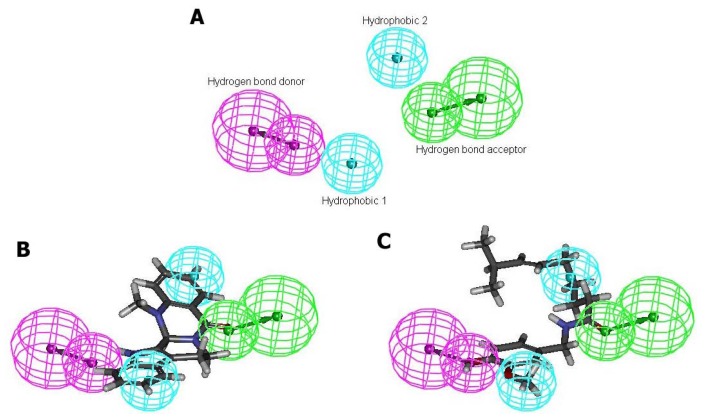
(**A**) Catalyst pharmacophore model (Hypo2) illustrating the hydrophobic regions (light blue), the hydrogen bond donor (purple), and the hydrogen bond acceptor (green); (**B**) Pharmacophore model Hypo2 aligned to evodiamine; (**C**) Pharmacophore model Hypo2 aligned to capsaicin.

**Figure 6:**
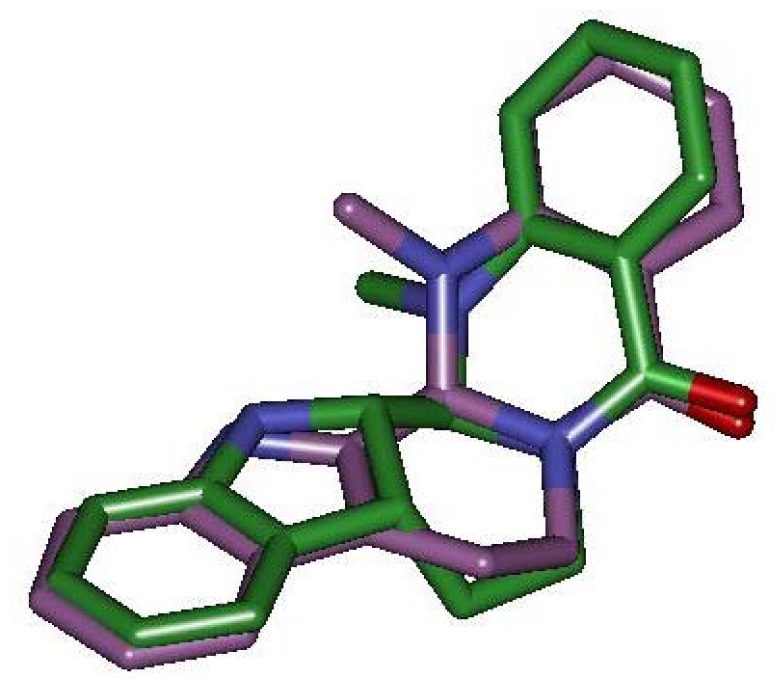
Docking conformation obtained for evodiamine (purple) was compared with that of the optimized one generated by Catalyst (green).

In order to understand why TRPV1 demonstrate species-specific sensitivity to capsaicin but not to evodiamine, four diverse TRPV1 agonists were taken to develop the pharmacophore model (Figure 1). Ten common feature hypotheses were produced. The second hypothesis (Hypo2) was selected for further analysis because it compiles the feature requirements of the training set considered as relevant (Figure 5a). The hypothesis Hypo2 is composed of four features: two hydrophobic groups, one hydrogen bond acceptor, and one hydrogen bond donor. On mapping of evodiamine it was observed that the molecule did map all the pharmacophoric features through critical TRPV1 recognition elements (Figure 5b). As expected, two hydrophobic groups (light blue) involve ring 1 and ring 5, the hydrogen bond acceptor (green) involves the formyl carbonyl oxygen, and the hydrogen bond donor (purple) involves the indole nitrogen. This binding motif is in general agreement with our docking studies. We also superimposed capsaicin on the Hypo2 (Figure 5c). Result indicated that capsaicin and evodiamine shared common pharmacophore elements. However, it was noticeable that capsaicin had relatively long and linear nonenyl tail compared with evodiamine, which was unable to map any feature of Hypo2 (Figure 5). Thus, capsaicin appeared to be inactive in rabbit TRPV1 due to steric hindrance between rabbit TRPV1 and nonenyl tail of capsaicin. This finding is in agreement with other studies [9]. Furthermore, the docked conformation of evodiamine was compared with that of the optimized one generated by Catalyst. A remarkable similarity was observed between these two conformations (rmsd: 1.892 Å) (Figure 6). This result is a significant confirmation for the reliability of pharmacophore model.

## 3. Experimental Section

### 3.1. Multiple Sequence Alignment

The primary sequence of TRPV1 in FASTA format for human (accession: Q8NER1), rat (accession: O35433), and rabbit (accession: Q6RX08) were retrieved from the UniProtKB database [21]. Because we focused on the transmembrane region, the sequences of the *N*- and *C*-terminal regions were removed. Multiple sequence alignment was performed with the ClustalW program [22].

### 3.2. Homology Modeling

We used the crystal structure of the voltage-dependent shaker family K^+^ channel available in the Protein Data Bank (PDB) 2R9R as the 3D coordinate template for the homology modeling of rat TRPV1. The sequence alignment of rat TRPV1 and the voltage-dependent shaker family K^+^ channel was carried out using the ClustalW program and manually refined to ensure alignment of important functional residues. The homology model of rat TRPV1 was constructed using MODELER program as previously described [11]. The initial model of rat TRPV1 was then embedded into a POPC lipid bilayer on a TIP3 water box considering the presence of counter ions. The entire system was submitted to a 5 ns MD simulation as previously described [20]. Human and rabbit TRPV1 homology models were built starting from rat model. Their initial models were refined using the same method as for rat TRPV1 model. The obtained structures were evaluated with the PROCHECK and ERRAT programs from the Structure Analysis and Verification Server [23,24]. Moreover, the tetramer models of TRPV1 were constructed according to the reported tetramer model [20].

### 3.3. Binding Site Analysis

The Binding Site tools in Discovery studio software (Accelrys, San Diego, CA, USA) allow researchers to calculate, edit, partition, and display binding sites of a receptor. There are two site finding routines that can be used to automatically locate binding sites. One identifies cavities within the receptor using an eraser algorithm [25], while the other builds a binding site based on the volume occupied by a known ligand pose already in an active site. In this study, the eraser algorithm was used to identify the possible binding sites within TRPV1 homology models. The standard default settings were used in the calculations except that minimum site size (points) was set to 150. The results can be used to guide the protein-ligand docking experiment.

### 3.4. Molecular Docking

The 3D structure of ligands were constructed and optimized in Discovery studio software (Accelrys, San Diego, CA, USA). Energy minimization was performed using MMFF94 force field and MMFF94 charge until the rms of the Powell gradient was 0.05 kcal/mol Å. Ligands were docked into the homology models using GOLD v3.0 [26]. The binding site was initially defined as all residues of the target within 10 Å from Leu515 (rat and human) and Leu518 (rabbit), and then automated cavity detection was used. The standard default settings were used in all calculations, and GOLD score was chosen to rank the hits. The resulting docked complexes were embedded into a POPC lipid bilayer, and solvated in a water box (TIP3 model) considering the presence of counter ions. The entire system was submitted to a 5 ns MD simulation as previously described [20].

### 3.5. Binding Energy Calculations

The free binding energy (Δ*G*_binding_) of a ligand was estimated according to the protocol described [27]. In brief, Δ*G*_binding_ was calculated as follows: Δ*G*_binding_ = Δ*G*_H_ + Δ*G*_EL_ + Δ*G*_S_ + *C*. The hydrophobic term Δ*G*_H_ is calculated with a 1.4 Å water probe, and multiplied by 30 cal/Å. The electrostatic term Δ*G*_EL_ is estimated with the continuum dielectric model with a protein dielectric constant of 8, water dielectric constant of 80, and probe radius of 1.4 Å. The entropic term was a sum of the side chain and ligand entropic terms. The constant value of 3 kcal/mol was used.

### 3.6. Pharmacophore Modeling

Ten diverse ligands from the available TRPV1 agonists were taken to develop the pharmacophore model (Figure 1). The compounds were built using the Catalyst 2D/3D visualizer in Catalyst 4.11 software package (Accelrys, San Diego, CA, USA) and are minimized to the closest local minimum using the CHARMm-like force field implemented in the program [28]. Catalyst generated a representative family of conformational models for each compound using the poling algorithm and the ‘best conformational analysis’ method [29]. Diverse conformational models for each compound were generated using an energy constraint of 20 kcal mol^−1^. The settings in conformer generation were 250 as the maximum number of conformers. The algorithm for hypothesis generation applied within Catalyst is termed HipHop. In this study, the chemical features used were hydrogen bond acceptor, hydrogen bond donor, and general hydrophobic features.

## 4. Conclusions

In summary, rat, human and rabbit homology models of TRPV1 were developed using the MODELER program. Using these validated models, evodiamine binding across various species was structurally characterized at the molecular level, which should assist us in the design of novel TRPV1 ligands.

## Supplementary Information
