# Supplementary material for: Binding Mode Prediction of Evodiamine within Vanilloid Receptor TRPV1
Source: Int J Mol Sci. 2012 Jul 18;13(7):8958–69. doi: 10.3390/ijms13078958 (PMC3430276; doi:10.3390/ijms13078958)

Binding Mode Prediction of Evodiamine within Vanilloid Receptor TRPV1

Supplementary Information

Figure S1. Ramachandran plots of the X-ray crystal structure of the voltage-dependent shaker family K+ channel (**a**), rat TRPV1 monomer model (**b**), human TRPV1 monomer model (**c**), and rabbit TRPV1 monomer model (**d**). The shading on the plot represents the different regions (red: the most favored regions; yellow: the allowed regions; beige: the generously allowed regions; white: the disallowed regions).

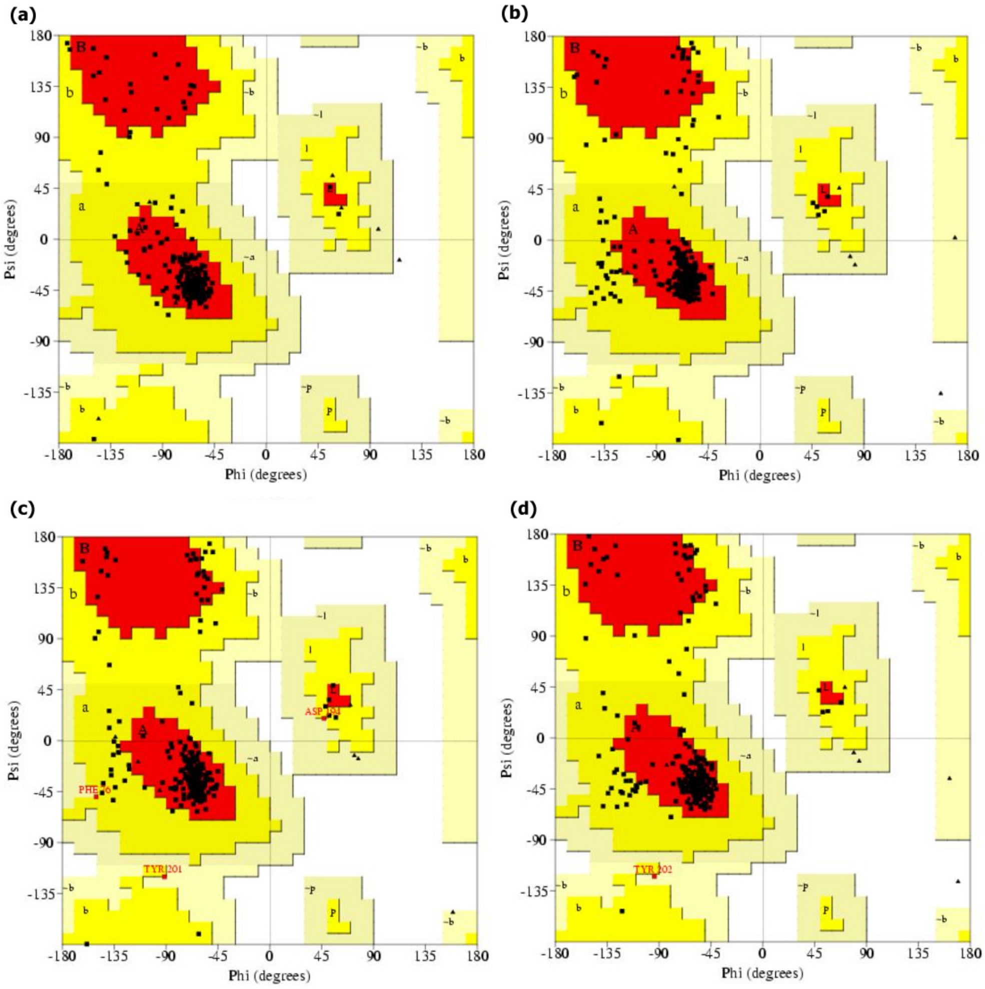

Figure S2. The possible binding-site of rat TRPV1 model. Site 1 represented by green color. Site 2 represented by yellow color. Site 3 represented by red color.

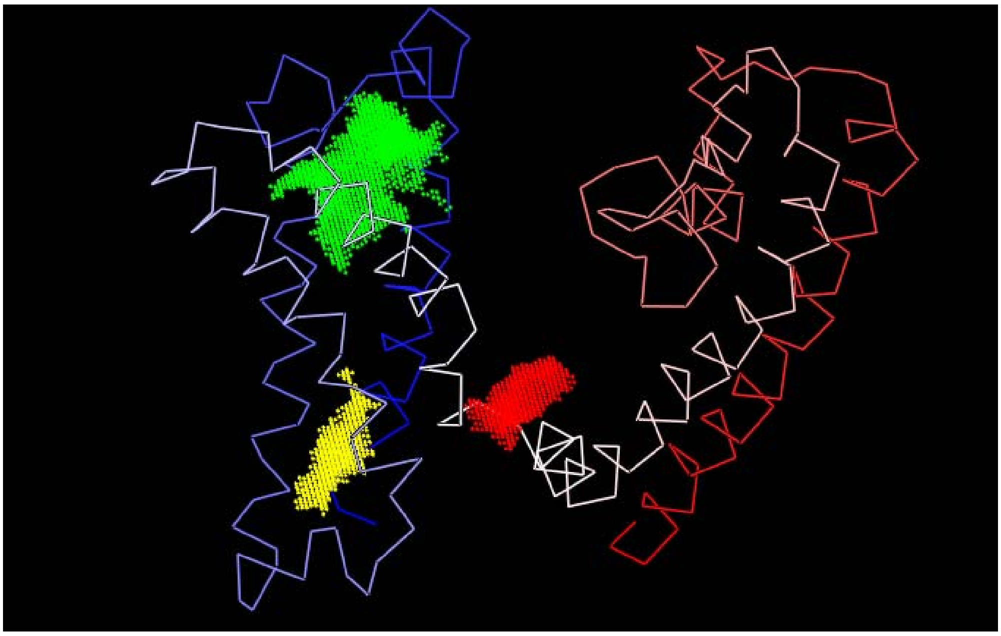

Figure S3. Overlay of the structures of rat TRPV1-capsaicin complex, human TRPV1-capsaicin complex, and rabbit TRPV1-capsaicin complex. The protein backbone is shown in ribbon view with rat TRPV1 in green, human TRPV1 in purple and rabbit TRPV1 in yellow. Capsaicin and the side chain of Thr550 (rat and human) and Ile553 (rabbit) are shown in stick representation with atoms colored to match their respective proteins.

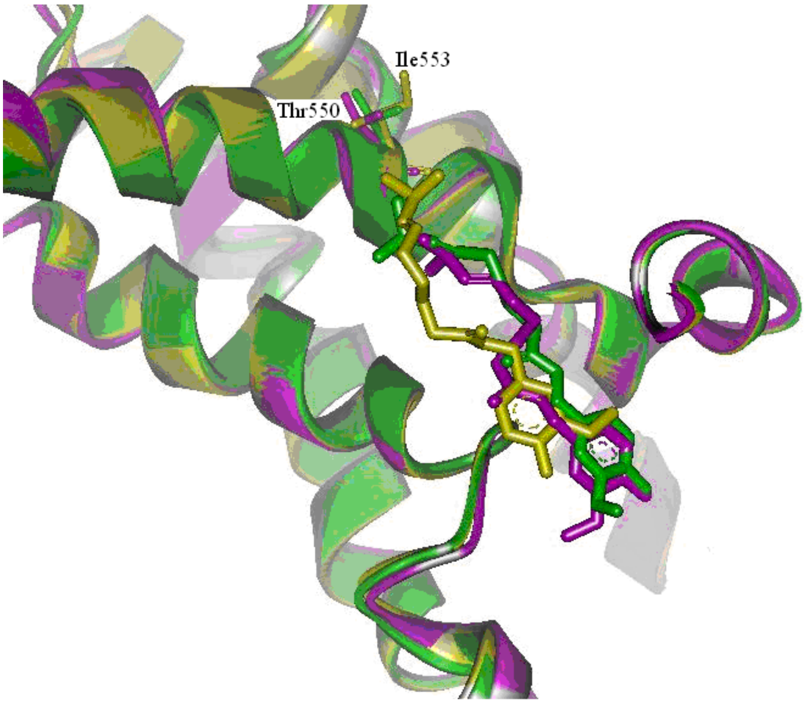

Supplement: Supplementary file 1 [file ijms-13-08958-s001.pdf]
